# Simultaneous Nanorheometry and Nanothermometry Using Intracellular Diamond Quantum Sensors

**DOI:** 10.1021/acsnano.3c05285

**Published:** 2023-10-04

**Authors:** Qiushi Gu, Louise Shanahan, Jack W. Hart, Sophia Belser, Noah Shofer, Mete Atatüre, Helena S. Knowles

**Affiliations:** Cavendish Laboratory, University of Cambridge, JJ Thompson Avenue, Cambridge CB3 0HE, United Kingdom

**Keywords:** thermometry, rheometry, biosensing, quantum sensing, nitrogen-vacancy center, nanodiamond, single particle tracking

## Abstract

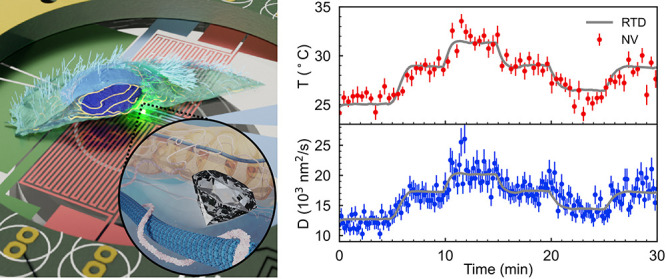

The viscoelasticity of the cytoplasm plays a critical role in cell morphology, cell division, and intracellular transport. Viscoelasticity is also interconnected with other biophysical properties, such as temperature, which is known to influence cellular bioenergetics. Probing the connections between intracellular temperature and cytoplasmic viscoelasticity provides an exciting opportunity for the study of biological phenomena, such as metabolism and disease progression. The small length scales and transient nature of changes in these parameters combined with their complex interdependencies pose a challenge for biosensing tools, which are often limited to a single readout modality. Here, we present a dual-mode quantum sensor capable of performing simultaneous nanoscale thermometry and rheometry in dynamic cellular environments. We use nitrogen-vacancy centers in diamond nanocrystals as biocompatible sensors for *in vitro* measurements. We combine subdiffraction resolution single-particle tracking in a fluidic environment with optically detected magnetic resonance spectroscopy to perform simultaneous sensing of viscoelasticity and temperature. We use our sensor to demonstrate probing of the temperature-dependent viscoelasticity in complex media at the nanoscale. We then investigate the interplay between intracellular forces and the cytoplasmic rheology in live cells. Finally, we identify different rheological regimes and reveal evidence of active trafficking and details of the nanoscale viscoelasticity of the cytoplasm.

Nanorheology addresses the question of how soft materials deform and flow at the nanoscale.^1,2^ Of significant interest in nanorheology is the study of complex cellular media such as the cytoplasm, which heavily influence cellular processes such as transport,^3^ division,^4,5^ and morphological changes.^6^ These properties, like many others in the cell, are linked to local biochemical energetics where temperature plays a critical role.^7,8^ It is well-established that cells regulate their viscoelastic properties in response to external temperature changes through homeoviscous adaption^9,10^ and viscoadaption.^11^ Variations in intracellular temperature, rheology, and their interdependence at the nanoscale remain outstanding questions today^12,13^ in the pursuit of a deeper understanding of cellular homeostasis, disease progression,^14^ and pathways for cancer treatment.^15^ The current challenges for existing biosensing tools include small length scales and a poor signal-to-noise ratio of the phenomena under investigation.

Optical techniques can provide means for investigating intracellular phenomena at the nanoscale in a noninvasive way. These methods are often susceptible to variations in autofluorescence,^16,17^ spectral transmission,^18^ and refractive index,^19,20^ which are typically present in complex biochemical environments. The interdependence of physical properties in biological systems can also be obfuscated by local inhomogeneity. Further, a change in one property, for example, temperature, can often affect others such as viscosity, the speed of chemical reactions, or the rate of cell division. The relationship between two properties is thus hard to capture effectively if the level of an external perturbation cannot be measured accurately and independently. Multimodal sensors offer the opportunity to reveal such interdependence.

Among the many approaches to nanoscale sensing in biological systems that are currently being explored, nanoparticles provide a platform which enables robust optical intracellular sensing.^18,21−23^ Nanodiamonds containing nitrogen-vacancy centers (NV) are one of the leading candidates: their properties include stable photoluminescence (PL), minimal cytotoxicity at high concentrations,^24,25^ amenability to surface functionalization,^26^ and robustness against changes in pH.^27^ The ground-state spin transition that is utilized for sensing can be effectively uncoupled from background fluorescence fluctuations, enabling NV measurements to be unaffected by local changes in the optical environment. The NV has the capability to measure several different quantities, as demonstrated separately for temperature,^28^ magnetic field,^29^ electric field,^30^ pressure,^31^ reactive oxygen species,^32^ and through targeted surface functionalization, pH.^33^ These demonstrations position the NV as a promising candidate for multimodal sensing implementations.

In this work, we perform nanothermometry and nanorheology using optically detected magnetic resonance (ODMR) and particle tracking of NV-containing nanodiamonds. We first demonstrate the operational protocol and achieve 3.7 nm spatial resolution with 9.6 ms update rate and a temperature sensitivity of . We quantify the performance in multiple well-controlled fluidic environments and then employ our sensor inside live human cancer cells and reveal different regimes of intracellular dynamics, while simultaneously measuring temperature. This dual-modality sensing is performed on a custom biosensing chip capable of microscopic temperature control and coherent spin manipulation.

## Results and Discussion

### Calibrating the Sensor Performance of Nanodiamonds

**Figure 1:**
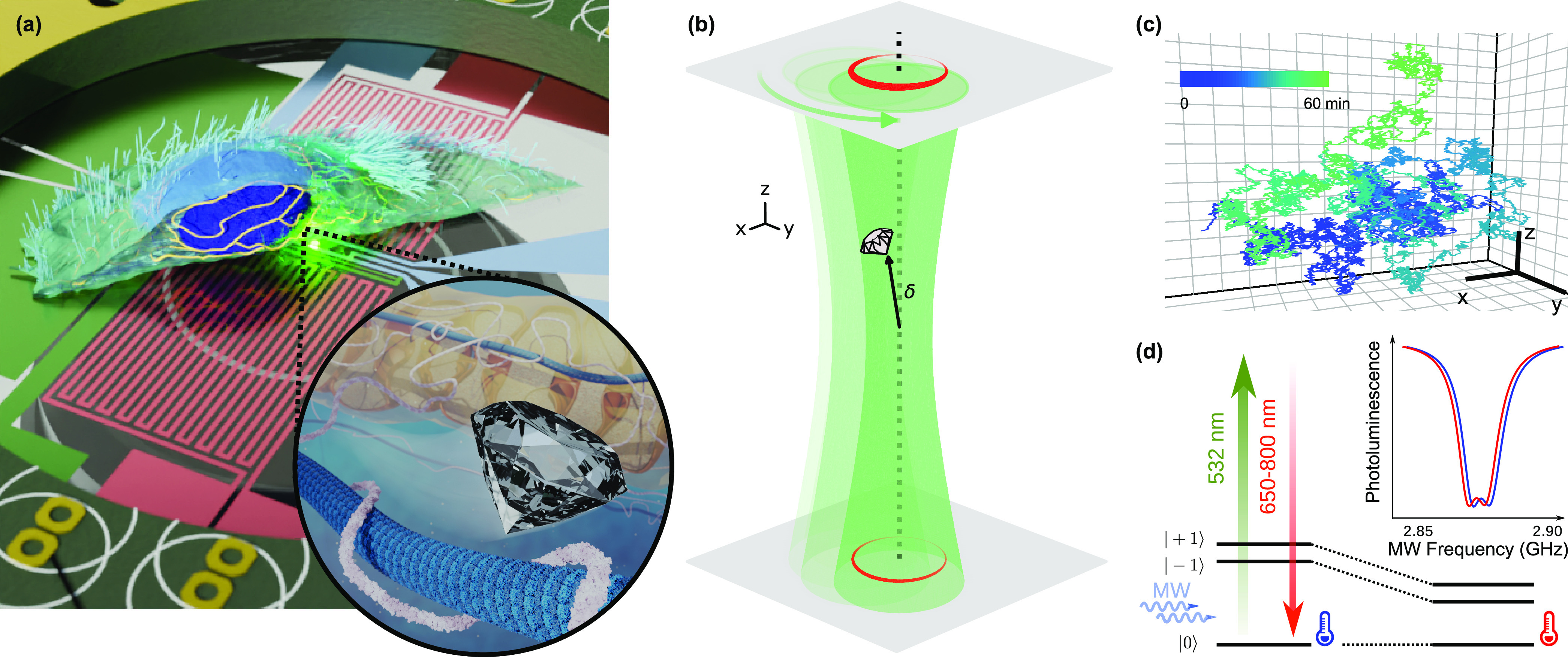
Diamond-based nanothermometer and nanorheometer. (a) An illustration of the cross-section of a cell grown on a custom sensing chip, consisting of a resistive temperature detector (blue), two resistive heaters (red), and a coplanar waveguide (green), which is used for accurate temperature control and microwave delivery. Inset: A nanodiamond interacts with its complex surroundings in the cytoplasm, including the microtubules (blue), actin filaments (pink), and mitochondria (yellow in background). (b) Real-time tracking is achieved by collecting the PL from a nanodiamond at two axially offset planes separated by 100 nm (red) as the excitation laser (green) orbits the last inferred position of the nanodiamond with a radius of 50 nm. Corrections (δ) in the transverse and axial directions are made to counteract any imbalance in PL along the orbit (indicated by the intensity of the red orbit) and between the top and bottom imaging planes (gray shaded planes). (c) An example trajectory of a nanodiamond undergoing Brownian motion in glycerol. The background grid has a spacing of 1 μm. (d) The transition frequencies of the NV ground state are temperature dependent and probed using ODMR (top right), with the central frequency of the ODMR spectrum decreasing with increasing temperature (blue to red).

To achieve optical readout of the NV spin, which underlies the sensing concept of nanodiamonds, we use a home-built confocal microscope (Supporting Information Section 1). Figure 1a and its inset illustrate the experimental arrangement, where a nanodiamond moves inside a cell while sensing local temperature. We use nanodiamonds that contain an ensemble of 100–300 NVs, with a radius of ∼25 nm. Nanoparticles of comparable sizes move with diffusion coefficients exceeding 3 × 10^4^ nm^2^ s^–1^ in cells^34^ (Supporting Information Section 2). These dynamic environments require the nanodiamond to be tracked throughout the optical spin readout measurements. We achieve this through a double-plane orbital tracking method,^35^ which provides real-time feedback control of the nanodiamond’s location, as illustrated in Figure 1b. The excitation laser performs circular orbits in the transverse plane with a period of 9.6 ms. The two confocal collection planes are offset symmetrically by ∼50 nm in opposite axial directions from the laser focus and collect the NV PL along the two offset circular paths. The asymmetries in PL around the orbit and between the top and bottom planes provide feedback parameters in the transverse and axial directions, respectively, updating the center of the orbital tracking to the nanodiamond position (Supporting Information Section 3). In Figure 1c we demonstrate the tracking of such a nanodiamond diffusing in glycerol.

While tracking the nanodiamond in real time, we simultaneously perform continuous-wave ODMR for temperature sensing. The ground state zero-field splitting of the NV can be optically read out by driving the NV spin from the *m*_s_ = 0 state to the *m*_s_ = ± 1 states. On resonance, this leads to a decrease in PL, as shown in Figure 1d. These transition frequencies are dependent on temperature. We sweep the microwave frequency over the target range every ∼1 ms and monitor the NV PL continuously to identify the spin resonances. We infer the change in temperature from the change in the central frequency of the full ODMR spectrum. The central frequency is extracted using an interpolation method (Methods, Supporting Information Section 4).

The ODMR-based thermometry technique requires the delivery of microwaves to the region of interest. This typically leads to heating of the substrate and intracellular medium. These effects can be a challenge to control and may vary from sample to sample. To achieve reproducible temperature control, sample heating with minute-scale temporal resolution (Supporting Information Section 5) and microwave delivery for the manipulation of NV spins, we developed a custom fabricated chip. All measurements are performed using a gold-patterned glass coverslip comprising a coplanar waveguide, two resistive heaters and a resistive temperature detector (RTD), as highlighted in Figure 1a with green, red, and blue regions, respectively. A polydimethylsiloxane (PDMS) open-top well is incorporated into the sensing chip to contain liquid samples when necessary.

**Figure 2:**
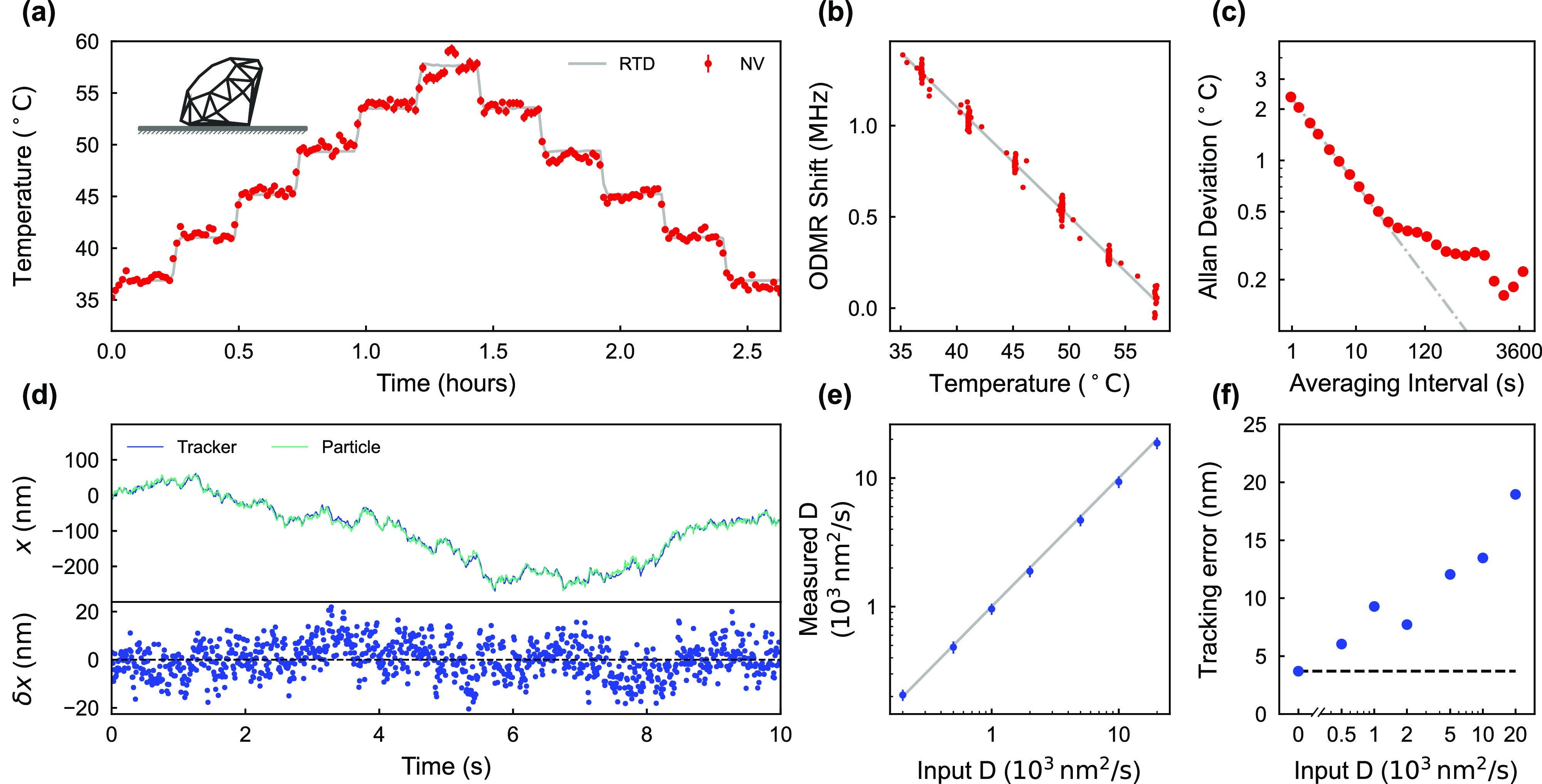
Accuracy and precision of the nanodiamond nanothermometer and nanorheometer. (a) The substrate temperature (gray curve) is stepped by 4 °C every 15 min, with the corresponding temperature reported by NV ODMR (red data). (b) The frequency shift is proportional to the change in temperature, with a temperature dependence of κ = −60.0 ± 0.4 kHz/°C. (c) The temperature precision over an accumulation time is characterized by the Allan deviation, from which we extract a sensitivity of . (d) Comparison between the known position (cyan data) in the *x*-direction of a nanodiamond moved in a Brownian motion-manner and the tracker-reported position (blue data), with the corresponding difference (*δ x*) shown in the lower panel. The set diffusion coefficient is 2 × 10^3^ nm^2^/s for this measurement. (e) The measured diffusion coefficient using the mean square displacement (MSD) at a time interval of 1 s shows a close agreement with the input diffusion coefficient. (f) The dynamic tracking accuracy, which is the standard deviation of the discrepancy between the tracker and particle trajectory, depends on the diffusion coefficient. When the particle is stationary, our system has a benchmark spatial resolution of 3.7 nm with 9.6 ms update rate (black dashed curve).

To benchmark the thermometry modality, we first quantify the temperature sensitivity of a stationary nanodiamond drop-cast on the quantum sensing chip in the absence of any fluidic environment. As seen in Figure 2a, the substrate temperature is adjusted in steps of 4 °C and the ODMR central frequency shifts proportionally. We extract a temperature dependence of κ = −60.0 ± 0.4 kHz/°C as shown in Figure 2b. Using the Allan deviation, Figure 2c shows an extracted sensitivity of  which agrees with the shot noise-limited sensitivity as predicted by the Cramer-Rao bound, , to within 10% (Supporting Information Section 7).

To benchmark the rheometry modality, we start by verifying the dynamic tracking accuracy of the single-particle tracking method. The scanning mirrors and the objective lens are moved such that a stationary nanodiamond on the substrate exhibits a predefined trajectory that mimics Brownian motion (Methods, Supporting Information Section 8). Figure 2d demonstrates the difference between the tracker trajectory (blue data) and the predefined particle readout (cyan data) over a 10 min interval which is used to determine the tracking accuracy. To analyze the stochastic diffusive motion, we compute the 2D mean square displacement (MSD), MSD(τ) = ⟨| **r**(*t* + τ) – **r**(*t*)| ^2^⟩, where **r** is the position vector in the transverse plane and τ is the time interval. The MSD depends linearly on the time interval for a particle undergoing Brownian motion, as MSD = 4*Dτ*, where *D* is the diffusion coefficient. The measured diffusion coefficient agrees with the input diffusion coefficient as highlighted in Figure 2e. In our system, we reach an upper bound of *D* = 5 × 10^4^ nm^2^ s^–1^, exceeding the typical intracellular diffusion coefficients observed with similarly sized nanodiamonds (Supporting Information Section 2). When the particle is stationary, we measure a resolution of 3.7 nm with a 9.6 ms update rate, as shown in Figure 2f, which is ∼60 times smaller than the 250 nm radius defined by the 1/*e*^2^ point-spread function. Our particle tracking is capable of following nanodiamonds in a range of dynamic environments with a high enough velocity and spatial resolution to allow the extraction of viscoelastic moduli. The nanodiamonds simultaneously operate as quantum sensors for temperature without the need for measurement deadtime in either modality.

### Dual-Modality Nanosensing in a Viscosity-Tunable Fluid

**Figure 3:**
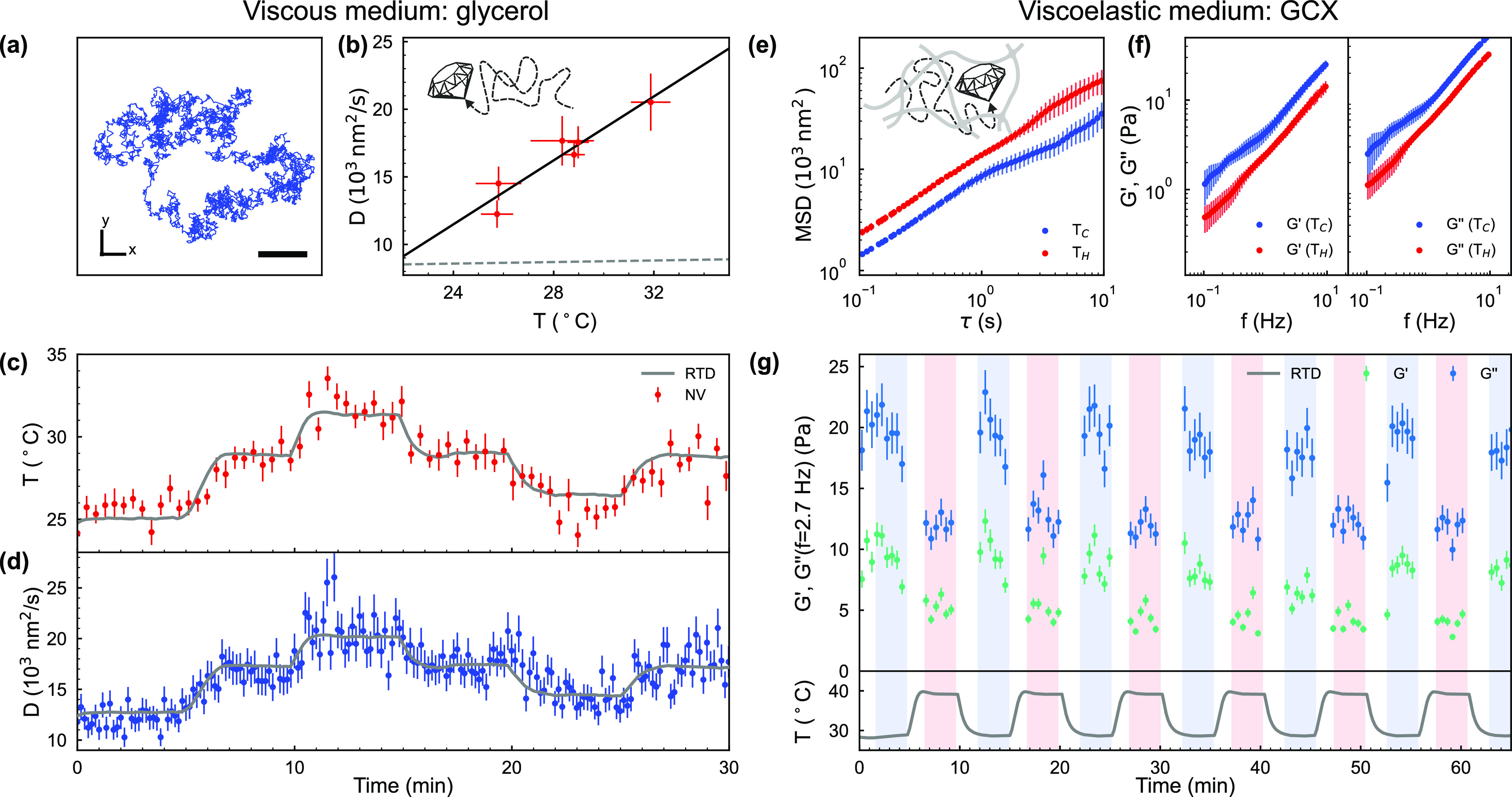
Temperature and rheology measurements in abiotic media. (a) An example of the nanodiamond trajectory projected onto the transverse plane over 96 s in glycerol. Scale bar: 1 μm. (b) The diffusion coefficient measured at different temperature values (red) with a linear fit (solid black curve) from which a hydrodynamic radius of 28 ± 1 nm is extracted. The gray dashed line shows the temperature dependence of the diffusion coefficient assuming a fixed viscosity of 0.919 Pa s corresponding to glycerol at 21 °C. (c, d) The simultaneous determination of temperature (red circles) and viscosity (blue circles) in glycerol, a purely viscous medium, measured using a single nanodiamond which was tracked for 30 min. The gray curve in (c) shows the temperature read out by the sensing chip and (d) shows the corresponding diffusion coefficient using the radius extracted from (b). (e, f) The mean square displacement (MSD) and viscous (*G*′′) and elastic (*G*′) moduli in a viscoelastic medium, glycerol-cross-linked xanthan (GCX), at *T*_C_ = 28.7 °C (blue circles) and *T*_H_ = 39.3 °C (red circles) obtained from nanodiamond tracking. (g) Temperature dependence of *G*′ and *G*′′ at *f* = 2.7 Hz for alternating temperatures *T*_C_ (blue shaded) and *T*_H_ (red shaded) as measured by the sensing chip (gray curve). (e and f) are calculated from the first 3 min of data at *T*_C_ and *T*_H_ in (g) (first blue and first red shaded regions).Data for panels a–d stem from one nanodiamond, data for panels e–g from a different single nanodiamond.

From the stochastic motion of nanoparticles, we infer properties about the surrounding material using passive nanorheometry. This provides a quantitative description of the relationship between nanodiamond motion and active forces. To demonstrate the use of nanorheometry with simultaneous nanothermometry, we studied nanodiamonds undergoing Brownian motion in glycerol. We choose glycerol as it can be assumed homogeneous and predominantly viscous and has a known temperature-dependent viscosity.^36^ In Figure 3a a particle is shown to travel several micrometers in 96 s. The particle motion is random, and thus, we use the MSD to extract the diffusion coefficient, *D*.

In glycerol *D* obeys the Stokes–Einstein relation, , where *T* is the absolute temperature, *k*_B_ is the Boltzmann constant, *r* is the hydrodynamic radius of the nanoparticle, and η(*T*) is the temperature dependent viscosity. We study the temperature dependence of the diffusion coefficient over a 17.5 °C range by increasing and decreasing the temperature in steps of 3.5 °C every 5 min. In the case of glycerol, η(*T*) is linearly dependent on temperature in the range probed, η(*T*) = η_0_ + μ(*T* – *T*_0_), with μ = 0.0208 Pa s/°C, *T*_0_ = 35 °C and η_0_ = 0.301 Pa s.^36^ From the experimental measurements of the diffusion coefficient in Figure 3 (b) (red data), we extract the temperature dependence of the viscosity, η(*T*) (black solid curve), using only the radius of the particle as a fitting constant. The estimated hydrodynamic radius of the nanodiamond is 28 ± 1 nm, which agrees with the nominal distribution provided by the supplier, 25 nm. As one would intuitively expect, a proportion of the diffusion coefficient’s temperature dependence can be attributed to increased thermal energy as seen in Figure 3b (gray dashed curve).

Figure 3c, d display the measured temperature, extracted from ODMR, and the diffusion coefficient, extracted from the nanodiamond trajectory, respectively. These nanoscale measurements were verified with the RTD-measured temperature and the corresponding extracted diffusion coefficient, respectively (gray curves). Using the multimodal sensor, we are able to probe the link between viscosity and temperature in glycerol through two simultaneous and independent measurements.

### Revealing Temperature-Dependent Viscoelasticity of a Complex Medium

In addition to sensing predominantly viscous rheological behavior, our probe can reveal the viscoelastic properties of biological environments such as DNA hydrogels^37^ and the actin cytoskeleton.^38^ To model these environments, we use the synthetic viscoelastic polymer network glycerol cross-linked xanthan (GCX). The complex modulus, G*(*f*) = *G*′(*f*) + *iG*′′(*f*), is used to characterize viscoelastic materials and can be calculated from the MSD,^39,40^ where *f* is the frequency of external perturbations at which G is measured. The real part of the complex modulus, *G*′(*f*), is a measure of the elasticity of the material and the imaginary part, *G*′′(*f*), is a measure of the viscous component. The ratio of the real and imaginary parts of the complex modulus establishes whether an environment is dominated by viscosity or elasticity. This capability can be used to capture how the rheological properties of the medium react to external perturbations.

Figure 3e displays the temperature-dependent MSD, obtained from the nanodiamond single-particle trajectory. In Figure 3f we demonstrate that |G*|, as well as its real and imaginary components decrease with temperature. This is the expected behavior for viscoelastic materials from the time–temperature superposition principle,^41^ as previously observed in other materials like hydrogels.^42^ We achieve sufficient sensitivity to distinguish between the two viscoelastic states with a 30 s averaging interval when we cycle the temperature by 10.6 °C. Figure 3g presents the change in viscous (blue) and elastic (cyan) moduli at two distinct temperature values of 28.7 and 39.3 °C.

### Capturing Signatures of Active Forces in Live Cells

**Figure 4:**
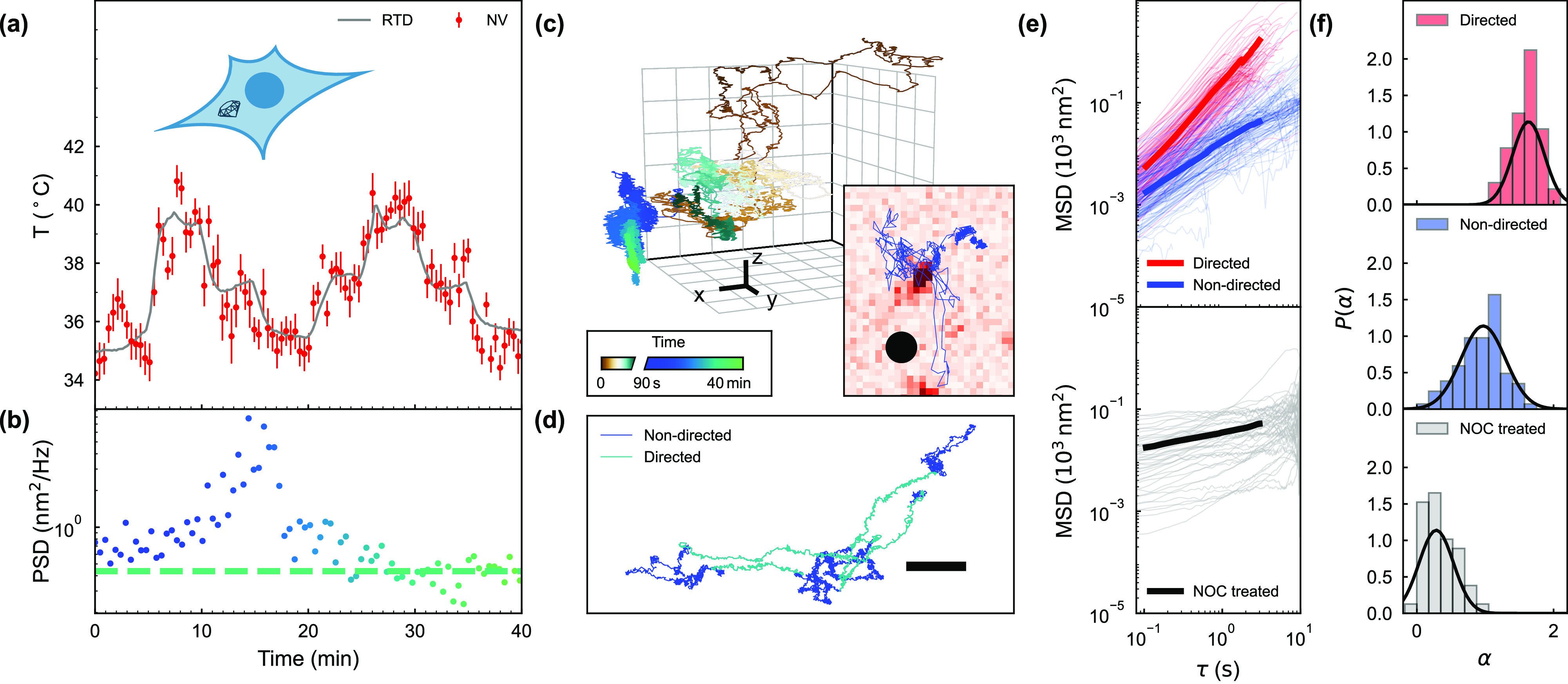
Nanodiamond multimodal sensing in live cells. (a, b) Simultaneous readout of temperature and power spectral density (PSD) in a cell. The gray curve in (a) shows the temperature read out by the sensing chip, and the PSD in (b) corresponds to *f* = 40 Hz. The dashed line represents the upper bound of the thermal contribution to the PSD. (c) Trajectory of a nanodiamond in a cell over 40 min xyz scale bar: 250 nm. Inset: xy particle trajectory relative to the optical diffraction limit (black spot, diameter = 500 nm). (d) xy particle trajectory showing both nondirected (dark blue) and directed (light blue) motion. Scale bar = 500 nm. (e) The mean square displacement, MSD, and ensemble averages (thick lines) for nanodiamonds with nondirected motion (blue) and directed motion (red) in untreated cells, and motion in cells treated with 50 μ M nocodazole for 1 h (gray). (f) Probability densities for the power-law exponents, α, for directed motion (red) and nondirected motion (blue) in untreated cells and cells that had been treated with 50 μM nocodazole for 1 h (gray). The black curves show fitted normal distributions.

Having benchmarked our dual-modal sensing approach in controlled environments, we next investigated the intracellular response to external temperature changes and the motion of nanodiamonds inside cells. We incubate HeLa cells with nanodiamonds and confirm internalization using 3D confocal microscopy (see Methods and Supporting Information Section 9). We expose the nanodiamond-containing cells to temperature cycles of 5.0 °C in steps of 2.5 °C lasting 5 min each. Figure 4a confirms the agreement of cell temperature measured independently by nanodiamonds (red circles) and the RTD temperature sensor on the sensing chip (gray curve). In this experiment, the nanodiamond was tracked and continuous temperature sensing was performed for 40 min, a significant advantage of the stable photoluminescence possible with nanodiamonds. Subsequent experiments performed on different nanodiamonds in live and fixed cells separately show no evidence of active temperature modulation by HeLa cells in response to external temperature modulations (Supporting Information Section 10).

Unlike glycerol and GCX, the cytoplasm of a cell is an active medium that is neither spatially homogeneous nor in thermal equilibrium. Molecular motors cause collective agitation of the cytoplasm.^3,43−46^ As such, particle motion represents the combined effect of both the material properties and the cellular activity. The power spectral density (PSD) of a particle’s location, ⟨*x*^2^(ω)⟩, which is the Fourier transform of the MSD, can be used to model this behavior. For media where the force–displacement relation is linear, this PSD is related to the power spectra of thermal stochastic forces, ⟨ξ^2^(ω)⟩ ∝ *k*_B_*T*,^3,43^ and active forces due to cell agitation, ⟨*F*_ext_^2^⟩, by Hooke’s law,
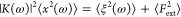
1

Here, *K*(ω) = (6*πr*) G*(ω) is the (complex) spring constant characterizing the property of the medium, G*(ω) is the complex modulus introduced in the previous section and ω is the angular frequency corresponding to the linear frequency, *f*. Figure 4b presents the PSD of the nanodiamond location averaged over 30 s at *f* = 40 Hz. The PSD increases dramatically approximately 15 min into the measurement, for a duration of around 10 min. Variations in the PSD can be explained by a combination of changes in |*K*(ω)| and ⟨*F*_ext_^2^(ω)⟩. Particular biological events such as cell division can result in large changes in viscoelasticity^4,5^ and thus changes in |*K*(ω)|. In the absence of such events, active microrheology^3,47^ and whole-cell AFM^48^ experiments suggest that cell viscoelasticity remains constant over the time scale of hours. The changes that we observe are therefore likely dominated by the active forces.

Nanodiamond internalization involves the endocytic pathway^49^ and therefore single-particle trajectories are expected to show temperature-dependent active trafficking^50^ together with the Brownian motion of the particle. As the nanodiamond spends the majority of the time in Brownian motion, the time-averaging used in the MSD and PSD analysis can hide transient features in the trajectory. Figure 4c shows the full trajectory of a nanodiamond in a cell over 40 min. We analyze this data by categorizing segments of nanodiamond trajectories according to periods of statistically significant directional persistence, characterized by the directionality ratio, γ = *d*/*l*, where *d* and *l* are the displacement and distance of a trajectory portion respectively (Supporting Information Section 11). Figure 4d shows an example of a nanodiamond trajectory containing directed motion segments. We compare the results from our segmentation method with the spread of anomalous diffusion exponents, α. Through the relation MSD ∝ τ^α^, the displacement behavior is typically classified into subdiffusive (α < 1), diffusive (α = 1) and superdiffusive (α > 1) states. Separating the trajectories into segments reveals that when the nanodiamonds are not in the directed motion state, they on average exhibit Brownian-like behavior, as can be seen from the MSDs in Figure 4e (top - blue) resulting in a power-law exponent of 0.97 ± 0.05 in Figure 4f (middle). In comparison, Figure 4e (top - red) and f (top), show that the nanodiamonds in the directed motion state appear superdiffusive, with a power-law exponent of 1.65 ± 0.05. The directed motion of the nanodiamonds could represent active trafficking around the cell interior. To investigate the effect of molecular motors, 50 μM of nocodazole was added to destabilize the microtubule network.^34,50^ Under this treatment, nanodiamond trajectories exhibited no directed motion. Further, the average power-law exponent of 0.3 ± 0.1 indicates subdiffusive motion as presented in Figure 4e (bottom) and f (bottom). From this we can infer that in the absence of active forces caused by microtubule-associated processes, the cytoplasm behaves as an elasticity-dominated weak gel^3,51^ (Supporting Information Section 12).

## Conclusions

Multimodal quantum sensing provides opportunities for investigating perturbation and response accurately and independently on the nanoscale in active biological environments. We identify the directed motion of the nanodiamonds as a possible indicator of active trafficking in the cell. Further, by removing the action of the molecular motors associated with microtubules, we show that the cytoplasm is dominated by its elastic properties. Our results also show that within our measurement sensitivity HeLa cells do not regulate their internal temperature in the presence of an external thermal perturbation.

The orbital tracking method we employ utilizes the stable photoluminescence of the NV and enables us to track single nanoparticles for over 40 min. This tracking method is not limited to the continuous-wave ODMR technique, and can be paired with more sophisticated quantum sensing protocols, such as nuclear magnetic resonance (NMR)^52^ or spin electron double resonance (SEDOR).^53^ Techniques such as optical tweezers^54^ and surface chemical functionalization^55^ can be combined with our dual-modal approach for precise localization of nanodiamonds with respect to subcellular organelles, such as mitochondria.^56^ The targeted delivery of nanodiamonds to subcellular regions would enable probing of potential hot spots, correlating local biochemical events with thermogenesis. This could be used to address the topic of nanoscale temperature gradients in live cells^57^ and be further extended to studying nonbiological soft matter. Active rheology techniques could allow further exploration of the relationship between active forces and the spring constants in cells. Combining nanodiamond sensors with super-resolution imaging techniques^58^ in a multiscale imaging setting offers an exciting opportunity to probe physical properties in the context of their biological environment.

## Methods

### Sample Preparation

#### Sensing Chip

The resistivity of gold at temperature *T*, *R*(*T*), is linear in the temperature range we probe and the measured RTD resistance is converted into temperature using
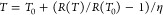
2where *T*_0_ is the reference temperature at which *R*(*T*_0_) is measured. *T*_0_ is measured with a thermocouple before each chip is used to calibrate the sensor. An experimentally determined fitting constant η = 2.44 ± 0.12 × 10^–3^ /°C is used in the conversion. A polydimethylsiloxane (PDMS) open-top well (with an approximate volume of 400 μL) is plasma-bonded to the substrate when performing experiments involving fluids.

#### Glycerol and GCX Suspension

To make a suspension of nanodiamonds in glycerol, 100 μL of 1 mg mL^–1^ 50 nm carboxylic-acid terminated nanodiamonds (FND Biotech, Taiwan) in water were centrifuged for 5 min at 12 000 *g*. Details on the nanodiamond fabrication and material properties can be found in ref (59). Excess water was removed, and the nanodiamond pellet was resuspended in 10 mL of ≥99% glycerol (Sigma-Aldrich, UK).

To make the glycerol-cross-linked xanthan (GCX), we transfer 9.9 g of glycerol-nanodiamond suspension prepared as mentioned above into a beaker and begin agitating by using a magnetic stirrer at a rate of 100 rpm. Then 0.1 g of xanthan gum powder (Sigma-Aldrich, UK) is slowly added to the glycerol. Once all the xanthan is fully incorporated into the glycerol, the beaker is heated to 80 °C with the stirring rate increased to 1000 rpm for 1 h to facilitate cross-linking.

#### Cell Preparation

We used the cervical cancer cell line HeLa as a model system for studying intracellular temperature changes. Prior to the experiment, HeLa cells (ATCC CCL-2) are incubated in an incubator at 37 °C with 5% CO_2_. To ensure cells adhere to the sensing chip, the substrate is coated in Geltrex basement membrane product (ThermoFisher Scientific, UK). Sufficient cell coverage was achieved by inoculating 10 μL of 1 × 10^6^ cells mL^–1^ in 390 μL of Dulbecco’s modified eagle medium (DMEM) supplemented with 0.11 g L^–1^ sodium pyruvate and 10% fetal bovine serum (FBS) directly into the PDMS well. Cells were left to adhere to the substrate for a minimum of 12 h prior to the addition of nanodiamonds.

To ensure an uptake of ∼2 nanodiamonds per cell, 1 μL of the nanodiamond stock solution was inoculated with the cells in the well for a minimum of 4 h. Excess nanodiamonds were washed off with phosphate-buffered saline (PBS). To compensate for the reduction in CO_2_ concentration during the ODMR experiments, the cells were inoculated in Leibovitz’s L-15 media (ThermoFisher Scientific, UK) supplemented with 10% FBS for all experiments.

To compensate for the temperature rise due to microwave heating, ODMR experiments were performed in an incubator (Digitalpixel, UK) maintained at 33 °C, which was given more than 12 h prior to the experiments to thermalize.

### Uptake Verification

We take particular caution in verifying the nanodiamonds are internalized inside the cells, to avoid measuring nanodiamonds attached to the surface of the cell. To achieve this, a series of 3D confocal scanning images on a Leica SP5 are taken to establish that the nanodiamonds are situated among the mitochondrial network which has been dyed with 100 nM MitoTracker Green FM (Sigma-Aldrich, UK) (Supporting Information Section 9). The observation that there are mitochondria above, below, and surrounding the nanodiamond observed confirms that the nanodiamond is internalized.

### Tracking System

We use a home-built confocal microscope for interrogating the nanodiamond fluorescence. The collimation lens on each collection arm is defocused in opposite directions so that one arm collects at a higher plane and the other at a lower plane than the laser focus. This is used for fast particle tracking in the longitudinal direction. Experimentally, we use a custom Teansy 4.0 microcontroller (MCU) for fast-feedback control. The photon counts are collected via a high-bandwidth counter, which are read out by the MCU. The fitting algorithm (implemented in Arduino IDE) uses a linear search and quadratic minimization algorithm. All computations are performed within 6 μ s.

The collection optics are separately aligned, and the collimation lenses are displaced in opposite directions so that the collected PL is 70% of the maximum.

#### Static Tracking Accuracy

The inferred trajectory of a stationary particle still appears moving. This results from intrinsic tracking error, setup drift, and other sources of movements. These noises are quantified by tracking a stationary nanodiamond (Supporting Information Section 8).

#### Dynamic Tracking Accuracy

Before the output voltage of the MCU control unit is applied to the actuators, it is additively combined with an external voltage, which can be either 0 V or a user-generated signal using an external signal source. The latter is used for characterizing the dynamic tracking performance. We generate voltages mimicking a Brownian motion particle and compare the extracted trajectory with the known trajectory to quantify the dynamics tracking accuracy.

### ODMR

For a 200-point sampling MW frequency ODMR spectrum, a full ODMR scan is completed in 2 ms. The scans are acquired for 160 ms (80 full scans) within a 200 ms duty cycle. The APD counters are gated such that they do not collect APD counts during the 40 ms of off time. The ODMR uses a global synchronization clock with 100 kHz frequency and gathers one PL reading on each clock edge.

During the 40 ms off time, the heater is switched on using a solid-state switch for 30 ms with a 5 ms buffer time before and after. The solid-state switch has a switching rise and fall time of approximately 0.5 ms. This avoids any magnetic field interfering with ODMR due to the current supplied to the on-chip heater.

### Data processing

#### ODMR Data Processing

The number of photon counts acquired from both APD1 and APD2 (shown in Supporting Information Figure S1) within 160 ms is read out (2 × 200 × 80 values, for two APDs, with 200 frequency points taken sequentially, and then 80 full repeated scans), converted to counts per second (by dividing by 10 μs), summed to give a total counts from both APDs, and stored as 80 × 200 values. Subsequently, each 80 rows of data (i.e., all data gathered within 160 ms time frame) is averaged (giving 200 values in counts per second) and stored as intermediate results for postprocessing. Subsequent data processing does not use the finely timed raw data due to the long processing time. For a typical emitter giving 1 Mcps emission, in 10 μs read out time, each APD reads about 5 photon counts.

### ODMR Data Fitting Methods

All data presented in this work use the interpolation method (Supporting Information Section 4). This method fits the entire data set using piece-wise linear interpolation to define an interpolation function and subsequently fits subsections of the data with this interpolation function to find any shifts in frequency, *δf*. These subsections are defined as independent bins of 400 ms ODMR data and result in a sequence of frequency shifts. We then average consecutive *n*_f_ frequency shifts to get a better estimate of the frequency shift. The error in the frequency shift is estimated using the standard error of the *n*_f_ frequency shifts considered. The resultant shift in frequency is converted into a temperature shift presented in the main text. See Supporting Information Section 4 for more details and motivation.

#### Computation of MSD and Associated Errors

We gather a sequence of 3D locations of the nanoparticle, denoted by **r**(*n*) = (*x*(*n*), *y*(*n*), *z*(*n*)), where *n* = 1, 2, 3, ..., *N* are the time indices. To compute the 1D time-averaged mean square displacement in the *x* direction, we compute the time average of the square of the pairwise differences, ξ_τ(*i*)_^*x*^ = *x*(*i* + τ) – *x*(*i*), by
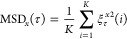
3where *K* = *N* – τ. As each *x* is a stochastic variable, the MSD is a stochastic variable with inherent variance, irrespective of measurement noise. To quantify this inherent stochastic variance, an estimator is needed based on the measured values of locations, considering the intrinsic correlation between ξ_τ(*i*)_^*x*^ and ξ_τ(*j*)_^*x*^ when | *i* – *j*| < τ as they are based on the same underlying trajectory data. We follow the approach in Kim et al.,^60^ in the limit τ ≪ *K* and *K* ≫ 1, where the variance of MSD_*x*_(τ) is estimated by
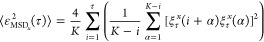
4Since there is no correlation between the three spatial directions
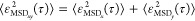
5The error bars in Figure 3d in the main text is computed using this method. It is estimated that in the limit where localization error is small compared to the stochastic variation due to Brownian motion, the error in determining *D* scales with . The exact proportionality constant depends on the specific method used.

We estimate the system noise floor to be at 10^–4^ μ m^2^, limited by the precision of the galvo mirror and signal sources used. The combined error is given by the noise floor if the statistical error derived above or the MSD itself is less than the noise floor.

#### Computation of Complex Modulus

At thermal equilibrium the complex modulus, G*(*f*) = *G′*(*f*) + *iG*^*″*^(*f*) = | G*(*f*)| *e*^*iδ* (*f*)^, is related to the MSD by,
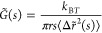
6where *G̃*(*s*) and ⟨Δ *r̃*^2^(*s*)⟩ are the Laplace transform of the complex modulus, *f* = 1/τ and the MSD with respect to the Laplace variable *s*. Using the method developed by Mason,^40^ this is related to the frequency dependence of *G* by
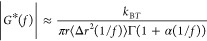
7where Γ is the Γ function. The loss tangent, δ, follows
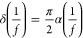
8Here  is the gradient of the MSD curve on a log–log plot. Error values for G* are calculated by propagating the MSD error through the above equation, assuming a comparatively negligible error on the fitted α value.

#### Power spectral density

The PSD, ⟨Δ*x*^2^(*f*)⟩ of motion in one axis was calculated using Welch’s method (welch function in the scipy.signal python module), averaged over 28.8 s. The total PSD is given by the sum of PSD in the two transverse axes: PSD = ⟨Δ*x*^2^(*f*)⟩ + ⟨Δ*y*^2^(*f*)⟩. PSD data presented in the main text are taken at *f* = 40 Hz.

## Supporting Information Available

Confocal microscope setup, diffusion constant statistics based on imaging experiments, double-plane orbital tracking, interpolation function, resistance temperature detector, experiment values for κ, mathematical limit of ODMR sensitivity, tracking performance, uptake studies, deviation in extracellular and intracellular temperature, track segmentation based on the directionality ratio and complex modulus analysis for nocodazole-treated cells (PDF)
