# Supplementary material for: Simultaneous Nanorheometry and Nanothermometry Using Intracellular Diamond Quantum Sensors
Source: ACS Nano. 2023 Oct 4;17(20):20034–42. doi: 10.1021/acsnano.3c05285 (PMC10604098; doi:10.1021/acsnano.3c05285)
Supplement: Supplementary file 1 — nn3c05285_si_001.pdf [file nn3c05285_si_001.pdf]

Supporting Information for 'Simultaneous Nanorheometry and Nanothermometry Using Intracellular Diamond Quantum Sensors'

Qiushi Gu,[†,‡] Louise Shanahan,[†,‡] Jack W. Hart,[†,‡] Sophia Belser,[†] Noah Shofer,[†] Mete Atatüre,[*,†] and Helena Knowles[*,†]

†*Cavendish Laboratory, University of Cambridge, JJ Thompson Avenue, Cambridge, CB3 0HE, United Kingdom*

‡*These authors contributed equally to this work.*

E-mail: ma424@cam.ac.uk; hsk35@cam.ac.uk

Supporting Information Section 1:

Confocal microscope set-up

The nitrogen-vacancy centers (NVs) are optically read out with a laser scanning confocal microscopy setup to achieve high spatial resolution. The confocal imaging part of the setup (Fig. S1) consists of an excitation arm and a collection arm. A 532 nm laser (Ventus 532, Novanta Photonics) provides the optical excitation. The laser is collimated after the single-mode fibre and overfills the back aperture of an oil immersion objective (Nikon CFI Plan Apo Lambda 100X Oil MRD01905, 1.45 N.A.). After collimation, 10% of the beam is split off to a photodiode (Si switchable gain detector, Thorlabs) for power monitoring. This

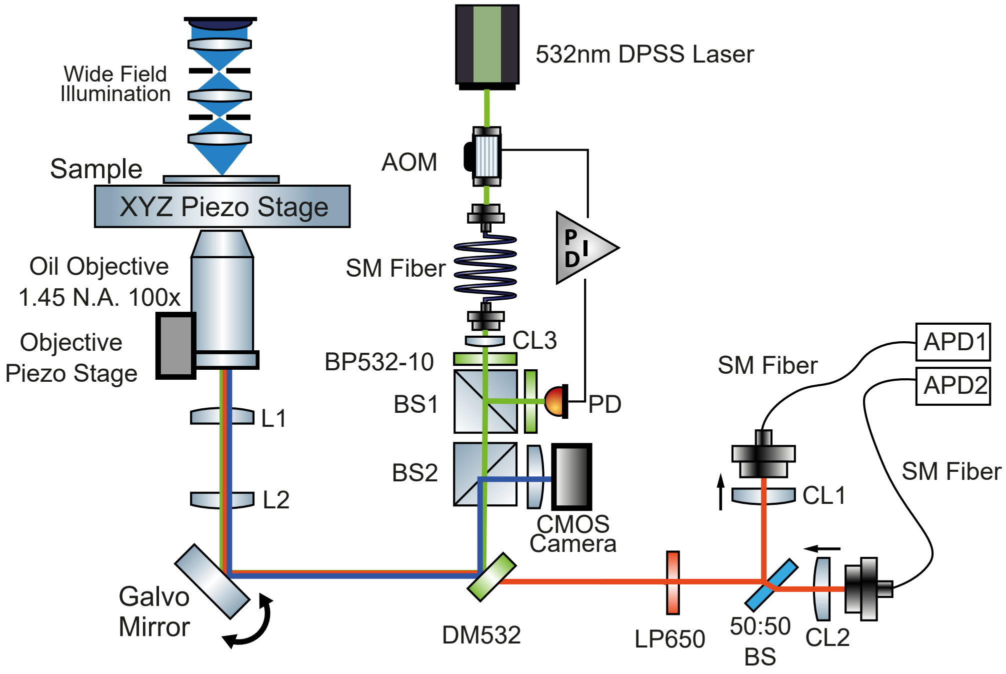

Fig. S1: Optical setup. A confocal set-up is used to excite the NVs with 532 nm and their fluorescence is collected between 650 nm and 800 nm. A 2D galvonomic mirror with a Keplerian telescope are used for xy positioning. The objective is mounted on a piezo-stage. The 2D galvonomic mirror and piezo-stage are used for single particle tracking. L1/L2: achromatic lens. BS1/BS2: nonpolarising beamsplitters. CL1/CL2/CL3: collimation lens. APD: Avalanche photodiode. SM Fiber: Single-mode fiber. AOM: Acousto-optic modulator. DM: Dichroic mirror. LP: Long-pass filter. BP: Band-pass filter.

power measurement is also used to stabilise the laser power via a high-bandwidth (100 kHz) PID controller (SIM900, Stanford Research Systems) which feeds back to a MT80-A1.5-VIS Acousto-optic modulator (Opto-Electronic).

The fluorescence from the NVs is collected through the objective and passes along the collection path where it is filtered by a 532 nm dichroic mirror, and a 650 nm long-pass collection filter, split into two collection pathways equally via a 50:50 plate beamsplitter, each focused via an achromatic lens into another single-mode fiber. Two avalanche photodiodes (APDs, SPCM-AQRH-14-FC, Excelitas technologies) are used for photon detection. The output pulses are counted by two DAQ cards (PCIe6321, PCIe6323, National Instruments) or by two custom high-bandwidth counters for the tracking system. All counter readings are synchronized to a global 100 kHz clock, generated by a DAQ card (PCIe6321, National Instruments).

The collimation lenses on each collection arm are defocused in opposite directions to 70% of the in-plane counts. This results in one arm collecting counts 50 nm above and the other 50 nm below the laser focus. This is used for fast particle tracking in the longitudinal direction. The digital computation takes $< 6\,\mu$s to complete and thus the feedback can potentially be reduced to sub-10 µs for faster moving particles if other parts of the system permit. A two-dimensional galvanometer mirror (GVS002, Thorlabs, UK) and an objective piezostage (DRV517, Thorlabs, UK) are used for feedback-based single particle tracking. The sample is mounted on an XYZ piezostage (NPoint, Inc., US) which provides a much larger range than the galvonomic mirrors and is used for slow sample positioning.

Supporting Information Section 2: Diffusion constant statistics based on imaging experiments

To verify that our tracking method does not significantly overlook the fast diffusing particles, we used a commercial scanning confocal microscope to build up the statistics for diffusion

constants. The tracking limit $D = 5 \times 10^4\,\mathrm{nm^2/s}$ covers 99% (Fig. S2) of all particles observed in a commerical confocal microscope.

Table S1: Diffusion speed of nanoparticles in other cells of human origin. The maximum speed tracked in this work ($0.05\,\mathrm{\mu m^2/s}$) exceeds all use case of single-particle tracking in individual cells.

Nanoparticle	Diameter	D_{max} ($\mathrm{\mu m^2/s}$)	Cell/Organism	Ref.
Polyplex	266 nm	0.0032	HuH-7 (human hepatome cells)	Dupont et al[1]
Polystyrene	110 nm	0.0003	Opossum kidney (OK) proximal tubule cells	Lanzano et al[2]
Quantum dot	-	0.03	Human lung carcinoma A549 cells	Li et al[3]

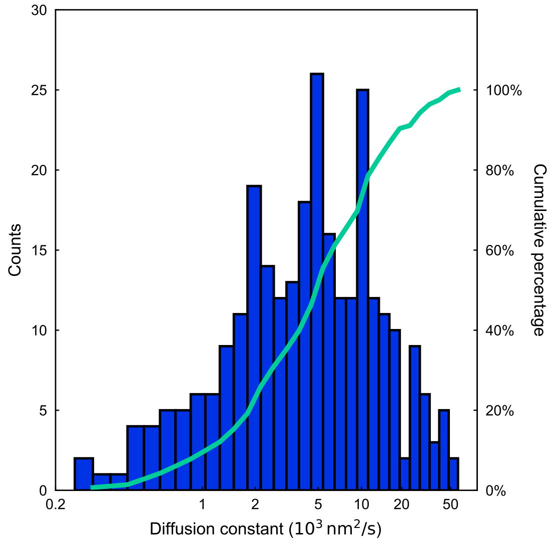

Fig. S2: Comparison between tracking performance and intracellular tracking requirement. Distribution of diffusion constants for nanodiamonds in HeLa cells extracted from image analysis based on confocal fluorescence images. The maximum tracking speed of our system, $D = 5 \times 10^4\,\mathrm{nm^2/s}$ covers 99% of all nanodiamonds observed, thus not inducing a bias.

Supporting Information Section 3: Double-plane orbital tracking

In the tracking process, the scanning mirrors are actuated to undergo a circular motion with radius R_{xy} and period T such that the deflection of each mirror at time t, can be expressed

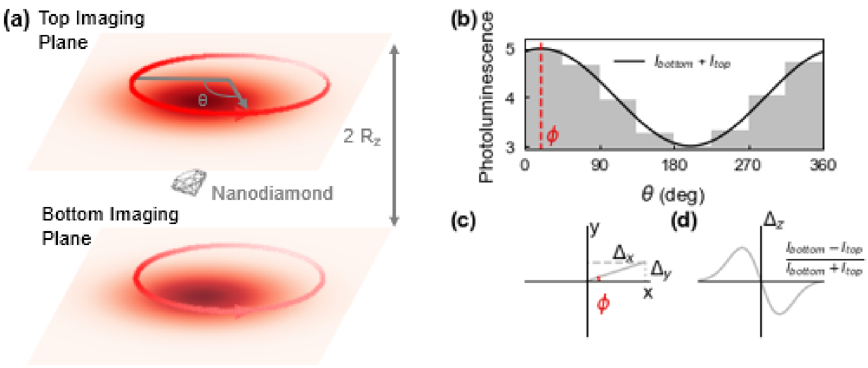

Fig. S3: **(a)** Orbital Tracking. Photons are collected from two imaging planes which are separated by $2\,R_z$. The tracker orbits the last known position of the nanodiamond. **(b)** and **(c)** The correction in the xy plane, Δ_x and Δ_y, are calculated by fitting the collected data (grey) to a sine wave (black). calculating the amplitude, $\delta \propto \sqrt{\delta_x^2 + \delta_y^2}$, and direction, ϕ, of the correction. **(d)** The axial correction, Δ_z, is calculated by finding the difference in counts between the top imaging plane and bottom imaging plane.

as

$$x = R_{xy} \cos\theta \tag{1}$$

$$y = R_{xy} \sin\theta \tag{2}$$

where $\theta = \frac{2\pi t}{T}$. The axial collection planes are displaced so that photons are collected from $z = \pm R_z$ simultaneously. The signals collected are binned into eight bins each corresponding to $\frac{1}{8}$ of the orbit. Thus, there are eight points per plane and two planes in total, giving 16 data points. These are at locations $(R_{xy}\cos(\theta_n), R_{xy}\sin(\theta_n), \pm R_z)$ where θ_n is the continuous angle $[n\pi/4, (n+1)\pi/4]$ for $n = 0, 1, \cdots, 7$. We denote the corresponding photo luminescence (PL) as, $I_{z,n}$ where $z =$ top denotes the top orbit and $z =$ bottom denotes the bottom.

As the confocal volume addresses hundreds of NVs, each of which is assumed to be randomly oriented, the point spread function (PSF) is assumed to be unaffected by the emission dipole orientation of the NV and of a Gaussian form, with w_{xy} and w_z being the $1/e^2$ radius of the PSF in the radial and the axial directions. For the confocal collection volume centered at (x, y, z) which orbits around a fixed point $(0, 0, 0)$ and a nanodiamond placed at $(\delta_x, \delta_y, \delta_z)$, the collected photon counts are,

$$I(\delta_x, \delta_y, \delta_z, x, y, z) = I_{\text{top/bottom,C}} R(\delta_x, \delta_y, x, y) Z(\delta_z, z), \tag{3}$$

where $I_{\text{top/bottom,C}}$ is the count rate on each collection arm when the emitter is at the point about which the confocal volume is orbiting and the radial and axial parts are,

$$R(\delta_x, \delta_y, x, y) = \exp\left(-2\frac{(x-\delta_x)^2 + (y-\delta_y)^2}{w_{xy}^2}\right), \tag{4}$$

$$Z(\delta_z, z) = \exp\left(-2\frac{(z-\delta_z)^2}{w_z^2}\right). \tag{5}$$

When the nanodiamond deviates from the focal spot by a small amount, $\delta_{x,y} \ll w_{xy}$,

$$R(\delta_x, \delta_y, x, y) \approx \exp\left(-2\frac{x^2 + y^2}{w_{xy}^2}\right)\left[1 + \frac{4x}{w_{xy}^2}\delta_x + \frac{4y}{w_{xy}^2}\delta_y\right] \tag{6}$$

Thus, along the circular orbit, the summed PL for the top and bottom axial planes is

$$I_n = I_{\text{top},n} + I_{\text{bottom},n} \approx I'\left[1 + \delta\cos\left(\theta_n + \phi\right)\right], \tag{7}$$

where $I' = [I_{\text{top,C}} Z(\delta_z, R_z) + I_{\text{bottom,C}} Z(\delta_z, -R_z)] \exp\left(-2\frac{R_{xy}^2}{w_{xy}^2}\right)$, $\delta^2 = (\delta_x^2 + \delta_y^2)/\varepsilon_{xy}^2$, $\varepsilon_{xy} = \frac{w_{xy}^2}{4R_{xy}}$ and $\tan\phi = \delta_y/\delta_x$. The parameters I', δ and ϕ are fitted using a least square fitting algorithm. An illustration of this process is show in Fig. S3.

As there are two collection planes going into two detectors, there is a slight mismatch between the collection readout due to differences in the fiber coupling efficiency, an unbalanced beam splitting ratio or misalignment in the optics. As a result $I_{\text{top,C}} \neq I_{\text{bottom,C}}$ and we require an additional term $G = \frac{I_{\text{bottom,C}} - I_{\text{top,C}}}{I_{\text{bottom,C}} + I_{\text{top,C}}}$ which is a setup dependent experimentally determined parameter. To compute the feedback parameter for the axial direction, the total PL from the top and bottom planes are $I_{\text{top,total}} = \sum_n I_{\text{top},n}$ and $I_{\text{bottom,total}} = \sum_n I_{\text{bottom},n}$. Then we compute the ratio,

$$r \equiv \frac{I_{\text{bottom,total}} - I_{\text{top,total}}}{I_{\text{bottom,total}} + I_{\text{top,total}}} \approx \frac{G - \delta_z/\varepsilon_z}{1 - G\delta_z/\varepsilon_z}, \tag{8}$$

where $\varepsilon_z = \frac{w_z^2}{4R_z}$.

After obtaining the parameters, δ, ϕ and r, we compute the corrections by,

$$(\Delta_x, \Delta_y, \Delta_z) = \left(\delta\varepsilon_{xy} \cos\phi, \delta\varepsilon_{xy} \sin\phi, \frac{r - G}{rG - 1}\varepsilon_z \right) \tag{9}$$

where $\Delta_{x,y,z}$ are the estimates of $\delta_{x,y,z}$ respectively. The parameters used are listed in Table S2. Note that the XY parameters are real experimental values.

Table S2: Parameter used for feedback localization locking.

Parameter	Value
T	9.6 ms
R_{xy}	50 nm
w_{xy}	260 nm
R_z	200 nm
w_z	200 nm
G	$[-0.2, +0.2]$

Supporting Information Section 4: Interpolation Function

Several lineshapes have been used to fit ODMR data including a double Lorentzian[4] and a single Lorentzian.[5] The double Lorentzian is a 7 parameter function and the single Lorentzian is a 4 parameter function. For an ODMR where the shape of the lineshape does not change, both of these fitting functions can be adjusted to have only 2 free parameters, the central frequency, f_0 and the counts, Λ_0. For ensembles of NVs the ODMR lineshape is a combination of the spectra from the individual NVs, each with their own central frequency and strain. As a result, the ODMR from ensemble NVs shows inhomogeneous spectral broadening and is not necessarily best described by a standard function.

We introduce the interpolation function as an alternative lineshape which can be used to fit ODMR data with two fitting parameters. For each pair of adjacent data points (f_1, PL_1)

and (f_2, PL_2) the linear interpolation function is defined by,

$$L^{\mathrm{Interp}}(f)|_{f_1}^{f_2} = \frac{1}{\Lambda_0}\left(\mathrm{PL}_1 + (f - f_1)\frac{(\mathrm{PL}_2 - \mathrm{PL}_1)}{(f_2 - f_1)}\right). \tag{10}$$

The lineshape function is then fitted to the data using

$$\mathrm{PL}(f) = \Lambda_0 L^{\mathrm{Interp}}(f - \delta f), \tag{11}$$

where the two fitting parameters are the off resonance PL, Λ_0, and the shift in frequency, δf.

To define the interpolation function we use the average over the full length of the ODMR dataset. This function was used to fit 400 ms of data and the offset in frequency was extracted. These frequency offsets are then further averaged so that each data point represents 12 s of data. The frequency offset is converted to temperature using the calibrated value for κ.

Comparison of interpolation function against other fitting methods

The interpolation function offers an advantage as it can be used to fit all lineshapes. In bulk NVs the intrinsic strain is usually quite low. However, in commercially available nanodiamonds there can be large variations in strain which can result in the presence of side peaks and/or assymmetry. The interpolation function is robust to the unusual ODMR lineshapes often seen in nanodiamonds. One caveat for using an interpolation function is that the data must be of a suitable length. As shown in Fig. S4 **(a)**, when the data is averaged over short periods of time the interpolation function retains noise from the data. The interpolation function is compared to the ‘true data’ which is the underlying lineshape from a nanodiamond before any noise was added to the system. Noise has been added using a gaussian

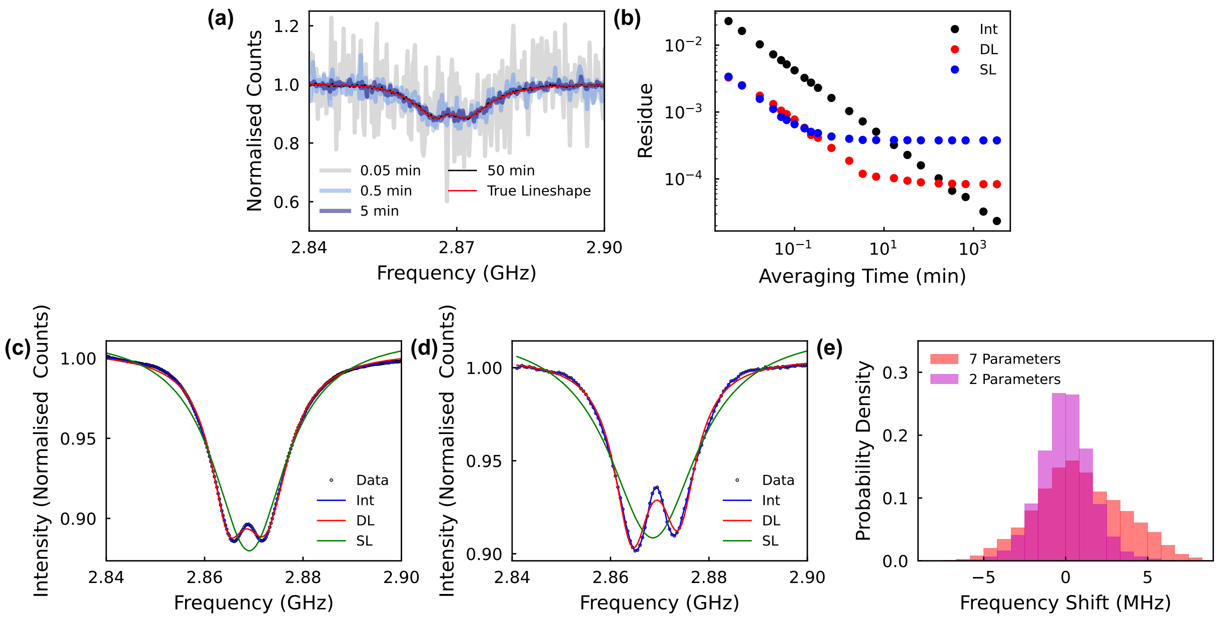

Fig. S4: Comparison of fitting lineshapes. **(a)** The noise in the interpolation function for different lengths of datasets. **(b)** The average residue in the normalised fit for the Interpolation function (blue), double-Lorentzian (red) and single-Lorentzian (green) for different lengths of dataset. **(c)** and **(d)** Finding the best lineshape for the dataset. Fitting the data (black) with a Interpolation function (Int - blue), double-Lorentzian (DL - red) and single-Lorentzian (SL - green). **(e)** Probability of achieving a frequency shift in the central frequency for a generated set of data with Gaussian noise at constant temperature. The double-Lorentzian fit was made with both 7 parameters (red) and 2 parameters (pink). The fixed parameters were extracted from the fitting in (c)

distribution with sigma calculated from the noise present on the nanodiamond. The average residue between the true data and the fitting model across the frequency range is calculated to quantify how well the function fits the data for a dataset of a certain length. As can be seen in Fig. S4 **(b)**, at short time intervals the interpolation function is dominated by noise but for longer time intervals the interpolation function more closely matches the true lineshape than the single Lorentzian ($T > 10\,\text{min}$) or the double Lorentzian ($T > 100\,\text{min}$).

In order to use a two parameter fitting method, it is important that the fitting function accurately represent the data. This involves both choosing the correct function for the lineshape and making sure any fixed parameters in the function are known. To find the fixed parameters, we average the data over the full dataset. This averaged ODMR is

then fit using all fitting parameters (7 for double Lorenzian, 4 for single Lorentzian) and the fixed parameters are extracted. Fig. S4 **(c)** shows a comparison for the different fitting models (Interpolation - blue, double Lorentzian - red and single Lorentzian - blue) against the averaged or true data (black). As can be seen in the figure, the intrinsic strain in the nanodiamond results in a splitting between the $m_s = \pm 1$ states and the single Lorentzian struggles to fit this. Fig. S4 **(d)** shows an example of an assymetric peak. We also take this opportunity to highlight the improvement in precision by using 2 parameter fitting. For a generated set of data at constant temperature with gaussian noise, the precision of the double Lorentzian 7 and 2 parameter fitting models were compared. As seen in Fig. S4 **(e)** the 2 parameter fit has a much narrower spread on the central frequency, 1.6 MHz than the 7 parameter fit, 2.7 MHz.

As described in section the theoretical sensitivity can be calculated for a given lineshape. For the data shown in Fig. S4 **(c)**, the theoretical sensitivities were found to be comparable for the interpolation function ($2.1\,\mathrm{K}/\sqrt{\mathrm{Hz}}$), double-Lorentzian ($2.2\,\mathrm{K}/\sqrt{\mathrm{Hz}}$) and single-Lorentzian ($2.0\,\mathrm{K}/\sqrt{\mathrm{Hz}}$) . As sensitivities are comparable and in this paper we work with long datasets, due to the robustness of the fitting model we use the interpolation model to fit the data.

Supporting Information Section 5: Resistance Temperature Detector

The operation of the Resistance Temperature Detector (RTD) is based on the linearity of electrical resistance of gold over the physiologically relevant temperature range. The resistance at temperature T is related to the resistance at reference temperature, T_{ref}, as (reproduced from Methods),

$$R(T)/R(T_{\mathrm{ref}}) = \eta(T - T_{\mathrm{ref}}) + 1, \tag{12}$$

where η is an experimentally determined constant related to the temperature coefficient of gold and is calibrated using the incubator. The on-chip heater and RTD allow the temperature to be controllably changed. The on-chip heater and RTD allow temperature to be changed by 2 °C within 2 min and the temperature stability can be maintained within 5 mK (data not shown). The positioning of the RTD relative to the CPW and heaters can be seen in Fig. S5.

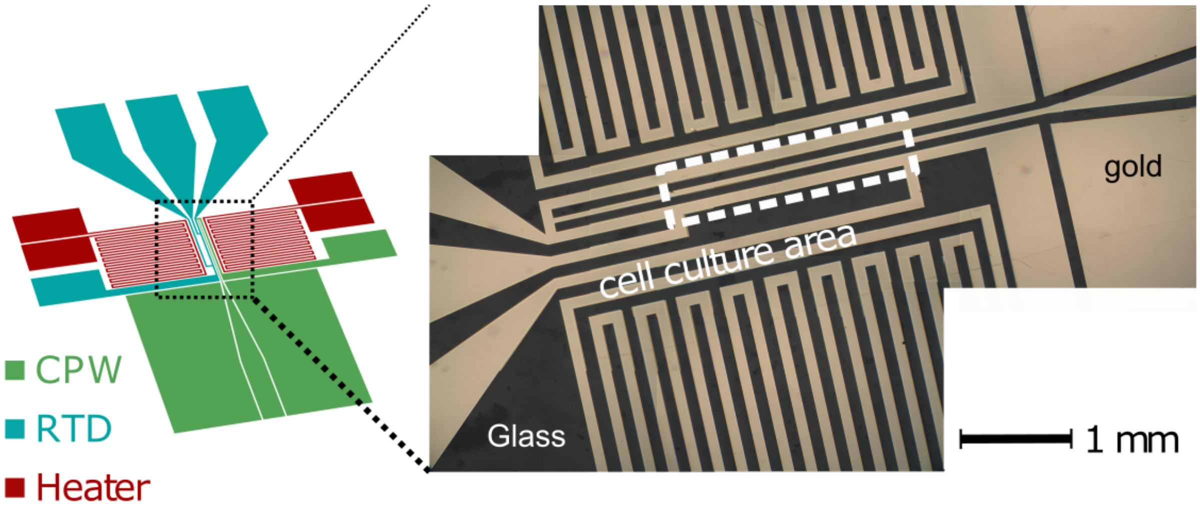

Fig. S5: Sensing chip. Left: schematic showing the different components of the pattern. Red: resistive heaters. Cyan: resistive temperature detector (RTD). Green: co-planar waveguide (CPW). In the micrograph on the right, the glass substrate is in black and gold pattern in yellow. The area where we choose to image is labelled with dashed rectangle, labelled “cell culture area".

Supporting Information Section 6:

Experiment values for κ

In Table S3, we provide a list of NV thermometry experiments reported to-date and the corresponding temperature-frequency conversion coefficients, κ, used in these works. Due to the differences in ODMR measurement methods and diamond manufacturer, the values vary significantly. When a constant value is assumed from the literature, or from a batch

calibration, the actual temperature may be under- or overestimated, thus causing systematic error.

Table S3: Values used to convert from ODMR central frequency shift to temperature change (κ). The typical cellular noise is derived from the ratio between the report spread and mean value of the biological process of interest.

$\lvert\kappa\rvert$ (kHz °C^{-1})	Cellular noise	Comments	Ref.
74	30%	Assumes constant value. Across multiple HeLa cells due to laser induced heating of surface dopamine.	Sotoma et al[4]
65.4	22%	Mean value of a few calibrated NDs. Temperature rise in multiple *C. elegans* due to chemical treatment with an uncoupler.	Fujiwara et al.[6]
74	8%	Assumes constant value. Across one neuron.	Simpson et al[5]
66 ± 11	-	Investigated κ dependence on chemical environment	Sekiguchi et al[7]
78 ± 12	-	Mean value of 15 NDs	Yukawa et al[8]
77	-	Assumes constant value	Kucsko et al[9]

Supporting Information Section 7: Mathematical limit of ODMR sensitivity

After obtaining the ODMR spectra, we use the nonlinear least-square curve fitting method to fit the data to a predefined lineshape function to estimate the shift of the ODMR central frequency. Various lineshape functions have been used previously, such as the double-Lorentzian[4] typically used in NV sensing based on single NVs and single-Lorentzian[5] functions. We wish to understand the theoretical limit of parameter estimation based on curve fitting, given the noise we see experimentally.

Here, we use the Fisher information to estimate the lower bound on the variance of estimated parameters, known as the Cramér-Rao bound (CRB). This is a technique widely used

in super-resolution imaging but has not yet been applied in the context of NV thermometry. Given a noise model (Poissonian for shot-noise limited measurements), a fitting function (for example double Lorentzian), and a set of points used to sample the fitting function (for example RF frequencies used to measure ODMR), CRB gives the lower bound on the uncertainty of estimation for any unbiased estimators.

To start with, we assume the experimentally measured ODMR spectrum consist of a sequence of measured values of photon counts in a unit time interval, $\mathrm{PL}(f_i)$ taken at various RF frequencies, f_i. These are random variables due to experimental noise, and follow the distribution

$$\mathrm{PL}(f_i) \sim \mathrm{Poisson}(\Lambda_0 L_\theta(f_i)), \tag{13}$$

where Λ_0 is interpreted as the off-resonance PL in a unit time interval and $L_\theta(f_i)$ is the lineshape function (normalised such that $L_\theta(f) = 1$ for non-resonant frequencies), parameterized by the constant vector θ. The lineshape function can be the linear interpolation function for which $\theta = (\delta f)$ (a single-element vector) where δf is the shift in ODMR central frequency. It can also be the double Lorentzian function in which case θ is a six-element vector with parameters characterising the contrasts, HWHM's and centers of each constituent Lorentzian. The CRB is applicable to any lineshape function with a variable number of parameters and so the following discussion is kept general.

The covariance matrix of the estimated values of θ, denoted by the estimator, $\hat{\theta}$, is given by the Cramér-Rao bound as,

$$\mathrm{cov}(\hat{\theta}) \geq \frac{1}{\Lambda_0} \left[\sum_i \frac{L'^2(f_i)}{L(f_i)} \right]^{-1}, \tag{14}$$

where $L' \equiv \partial_{\theta_i} L$ is the gradient of the lineshape function in the θ space and L'^2 is a square matrix with components $(L'^2)_{ij} \equiv (\partial_{\theta_i} L) \left(\partial_{\theta_j} L\right)$. Here, $i = 1, 2, 3, \cdots, N$ with N being the number of parameters used. The $(\bullet)^{-1}$ should be interpreted as the matrix inverse when θ has

more than one elements or the multiplicative inverse (in the algebraic sense) when θ has only one element. Two square matrices A and B satisfy the relation $A \geq B$ if $\langle i|(A-B)|j\rangle \geq 0$ for any choice of non-zero vectors $|i\rangle$ and $|j\rangle$. Specifically, if only the diagonal of the covariance matrix is of interest, then $A \geq B$ implies that every diagonal element of A is greater than or equal to that of B. Thus, by choosing the diagonal element corresponding to the central frequency, the CRB gives the lower bound on the uncertainty for frequency estimation. We use this to compute the theoretical sensitivity bound quoted in the main text.

Supporting Information Section 8: Tracking performance

Stationary tracking performance

The power spectral density (PSD)(Fig. S6) of the positions of a stationary particle reported by the tracker is computed using 190 s of tracking data in three spatial directions for each PL intensity corresponding to different laser power. In particular, we note that the static tracking noise is limited by the fluorescence (Fig. S6 (a) and (b)), such that increasing the fluorescence by using brighter particles or increasing the laser power can improve the tracking performance. A similar effect is shown in the PSD where the static tracking noise is also reduced with increased particle brightness, as shown in all axes in Fig. S6, (c)-(e).

Dynamic tracking accuracy

To characterize the dynamic tracking performance, an external, user-generated voltage signal can be additively combined with the output voltage of the micro-controller unit (MCU) before it is applied to the actuators. We use a DAQ card (PCIe 6323, National Instruments) to generate voltages that mimic a 3D random walk. For one dimension, such a random walk is

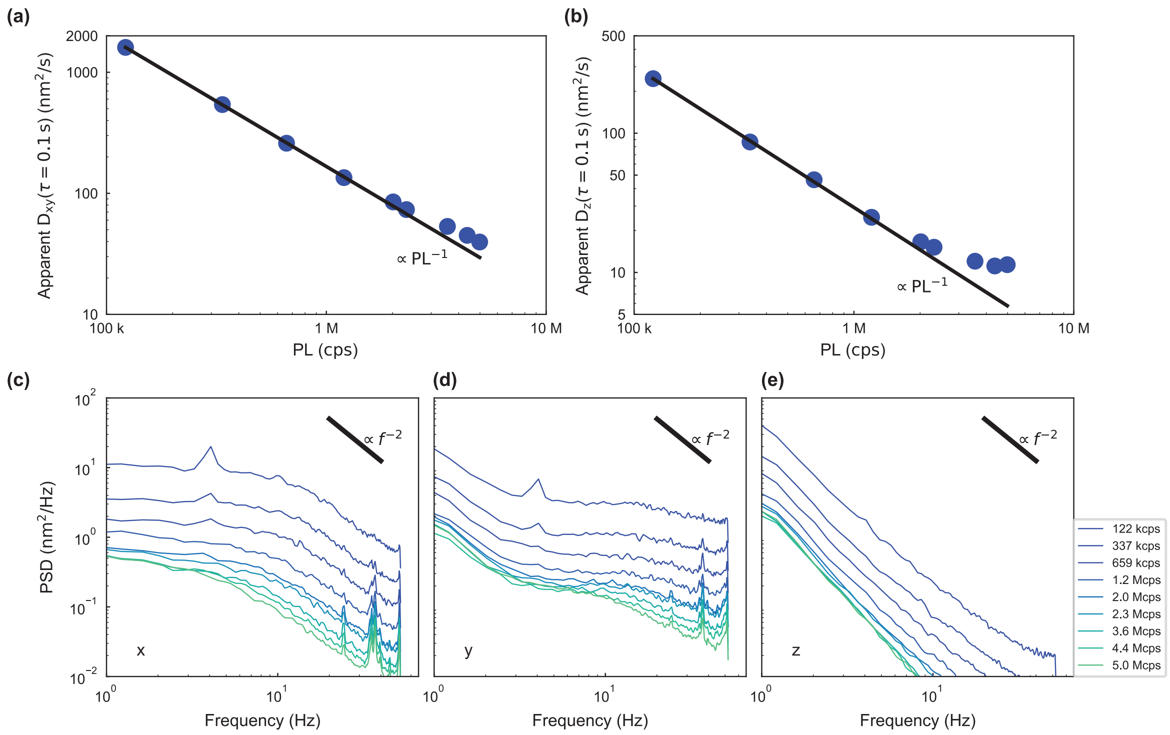

Fig. S6: Sensitivity of nanodiamond single particle tracking. **(a),(b)** The apparent diffusion constants, in XY and in Z direction, of a stationary nanodiamond, limited by photon-shot noise and other noise in the system. The apparent diffusion constant, D, scales with PL^{-1} for small PL emitters. **(c)-(d)** The power spectral density for individual axis.

given by,

$$x(n\tau) = \sum_{i=0}^{n} \Delta x_i, \tag{15}$$

where $\Delta x_i \sim \mathcal{N}(0, 2D\tau)$ is a sequence of randomly generated steps following a Gaussian distribution with a zero mean and a variance of $2D\tau$. D is the diffusion constant input by the user. The time step, τ, is set by the sampling rate of the signal source and is 240 µs. A different sequence of random numbers are used for each axis. The timing of the signal generator is again synchronized with the global 100 kHz clock so that the generated trajectory can be aligned temporally with data obtained from the tracking system or optically detected magnetic resonance (ODMR) experiments.

In principle, the signal generator and the tracking system are unaware of each other and thus

a nanodiamond fixed on a substrate would appear to be diffusing to the tracking system. The predefined trajectory is then compared with the extracted trajectory from the tracking system to measure the diffusion constant and localization accuracy (Fig. 2 (e)). As expected the dynamic localization accuracy is worse at high input diffusion rate, shown in Fig. 2 (f) in the main text.

Supporting Information Section 9: Uptake studies

We verify the nanodiamond internalisation by taking 3D confocal images of both the nanodiamonds and dyed mitochondria (Fig. S7 **(a)**). Nanodiamonds uptaken by the cell will be surrounded by the mitochondrial network in 3D (XY, YZ and XZ slices in Fig. S7 **(b)-(d)**). Before temperature measurements on the home-built confocal, we use the fluorescence from the cell culture medium to confirm the nanodiamond is in the cytoplasm in 3D (XY and XZ slices in Fig. S7 **(e), (f)**).

Supporting Information Section 10: Deviation in extracellular and intracellular temperature

In the main text we state that the intracellular temperature at the nanodiamond location over the timescale of the measurements equals the external temperature. Here, we give a detailed analysis of the statistical significance of this result.

In response to an external temperature rise, ΔT_{ext}, the potential effects of physical thermal shielding and active cellular response could cause a different internal temperature rise, ΔT_{int}. This would cause the ODMR frequency of an internalized nanodiamond to shift by less (more) than $\kappa_{\mathrm{NV}}\Delta T_{\mathrm{out}}$. A smaller (larger) effective $\kappa_{\mathrm{eff}} = \kappa_{\mathrm{NV}}\Delta T_{\mathrm{int}}/\Delta T_{\mathrm{ext}} \approx \kappa_{\mathrm{NV}} + \Delta\kappa_{\mathrm{eff}}$ would be measured.

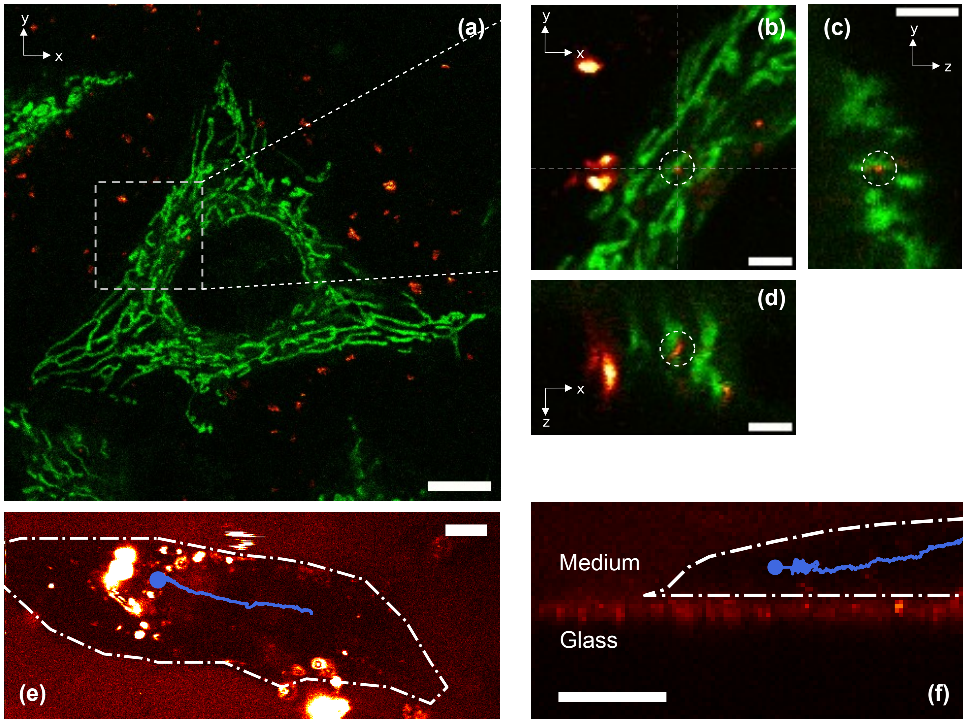

Fig. S7: Uptake verification using a laser scanning confocal microscope. **(a)** Two-color confocal image showing nanodiamond (red) internalization in a HeLa cell (where the image has been taken 500 nm above the bottom of the cell). Mitochondria (green) are used as a reference for identifying the cell's internal volume. Scalebar is 10 µm. Three-dimensional imaging was performed on the region highlighted by the dotted square. **(b)** Example image from the 3D stack taken 1.5 µm above the bottom of the cell. **(c)** Corresponding YZ projection and **(d)** XZ projection shown at the coordinates associated with the nanodiamond indicated by the dotted lines in (b). Mitochondria are visible above the nanodiamond confirming internalization. Scalebars in (a)-(d) is 3 µm. **(e)** XY confocal image of a HeLa cell taken on the ODMR confocal microscope prior to a temperature measurement. **(f)** XZ confocal image of the same cell. Scalebars in (e) and (f) are 5 µm. The blue curves represent the trajectory of the tracked nanodiamond and the white dashed curves represent the cell contour. XY is the imaging plane and Z is the optical axis direction in all Figures.

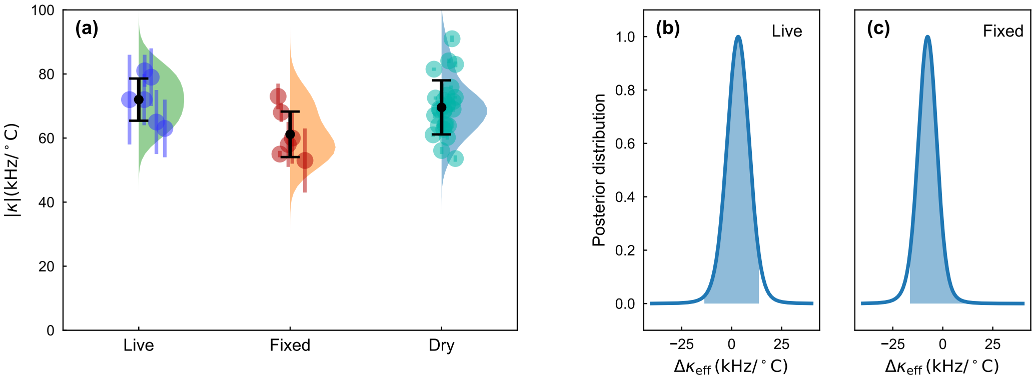

Fig. S8: Distribution of κ across nanodiamonds measured in different environments. **(a)** Variation of κ across different experimental conditions: live cells (blue data), PFA fixed cells (red data) and dropcast on a dry substrate (cyan data). The shaded region corresponds to the inferred statistical distribution using the kernel density estimation method. The black errorbars indicate the mean and standard deviation of the corresponding data. **(b, c)** Posterior distribution of $\Delta\kappa_{\text{eff}}$ for live and fixed cells given the prior distribution of dry sample variation and measurement uncertainty.

This measurement is in general confounded by the variation in κ between nanodiamonds. For example, although the nanodiamond presented in the main text Fig. 2 **(b)** has a proportionality constant of $\kappa = -60.0 \pm 4\,\text{kHz}/°\text{C}$, we measured a range from $-53.6 \pm 1\,\text{kHz}/°\text{C}$ to $-91.1 \pm 1\,\text{kHz}/°\text{C}$ in our samples of 26 nanodiamonds on dry substrates (cyan in Fig. S8 **(a)**). If a constant value of $-74\,\text{kHz}/°\text{C}$ is assumed, this variation can incur a $\pm 25\%$ systematic error, which highlights the need for a per-diamond calibration. The large intrinsic variation also implies that the cell-to-cell variation may be overestimated if a constant value of κ is assumed.

The intracellular response can be verified using statistical analysis. We measure the intracellular κ for a series of six experiments (blue in Fig. S8 **(a)**) in live cells and a series of six experiments in fixed cells (red in Fig. S8 **(a)**). Cells were fixed by incubating with 4% paraformaldehyde (PFA) in phosphate-buffered saline (PBS) at room temperature for 15 minutes.

As there are intrinsic variations between nanodiamonds, the naive combination of uncertainties in measured values of intracellular κ_{eff} and κ_{NV} is not sufficient to estimate that

of $\Delta\kappa_{\text{eff}}$. As such we adopt a Bayesian approach similar to Ref.[4] to obtain the statistical distribution of $\Delta\kappa_{\text{eff}} = \kappa_{\text{eff}} - \kappa_{\text{NV}}$, which incorporates uncertainties due to cellular noise, measurement uncertainty and intrinsic diamond variations. On the timescale accessible to our experiments, $\Delta\kappa_{\text{live}} = 3 \pm 13\,\text{kHz/°C}$ (Fig. S8 **(b)**) and $\Delta\kappa_{\text{fixed}} = -7 \pm 11\,\text{kHz/°C}$ (Fig. S8 **(c)**). As these deviations are not statistically significant, then it can be claimed that the cell does not actively compensate for external perturbations in temperature by inducing an internal temperature change.

Supporting Information Section 11:

Track segmentation based on the directionality ratio

To identify segments of the nanodiamond trajectory that have a statistically significant directionality ratio, γ, we first consider the motion of a particle undergoing Brownian motion in two dimensions. After a total of N steps, the distance travelled by the particle, l', will be equal to

$$l' = \sum_{t=1}^{N} \sqrt{\sum_{i=1}^{M} (\Delta r_{i,t})^2} \tag{16}$$

where $\Delta r_{i,t} \sim \mathcal{N}(0,1)$ and M = the number of dimensions (for a 2D projection, $M = 2$). The term inside the outer summation can be described by the variable, $Q_t \sim \chi(M)$, where $\chi(M)$ is the chi-distribution (namely the positive square root of the sum of squares of a set of independent random variables each following a standard normal distribution),

$$l' = \sum_{t=1}^{N} Q_t. \tag{17}$$

Now, using the central limit theorem, l' can be expressed in terms of the normal distribution,

$$\frac{l'}{\sqrt{N\sigma^2}} \sim \mathcal{N}(0,1) + \frac{\sqrt{N}\mu}{\sigma} \tag{18}$$

where μ and σ are the mean and standard deviation for $\chi(M)$ respectively. For the same particle, the displacement, d' can be described by,

$$d' = \sqrt{\sum_{i=1}^{M} \left(\sum_{t=1}^{N} \Delta r_{i,t} \right)^2} \tag{19}$$

which again can be characterized by the chi-distribution by normalizing by $\sqrt{N}$,

$$\frac{d'}{\sqrt{N}} \sim \chi(M) \tag{20}$$

The directionality ratio is defined as,

$$\gamma = \frac{d'}{l'}. \tag{21}$$

To find the probability density function for the directionality ratio, f_γ we must define a new variable, η, such that,

$$\eta = \frac{1}{\gamma}\sqrt{\frac{M}{\sigma^2}} \sim \frac{\mathcal{N}(0,1) + \frac{\sqrt{N}\mu}{\sigma}}{\frac{\chi(M)}{\sqrt{M}}}. \tag{22}$$

As this form is indicative of a non-central t-distribution with M degrees of freedom and a non-centrality parameter of $\frac{\sqrt{N}\mu}{\sigma}$, the probability density function for η, f_η can be ascertained analytically. As the total integrals for f_γ and f_η must equal 1, the function of f_γ can be found numerically,

$$f_\gamma = \left|\frac{d\eta}{d\gamma}\right| f_\eta = \frac{1}{\gamma^2}\sqrt{\frac{M}{\sigma^2}} f_\eta. \tag{23}$$

Regions of the nanodiamond trajectories were deemed statistically significant if the directionality ratio exceeded the critical value corresponding to a 5% probability of occurring randomly for a particle undergoing Brownian motion. For the experimental data, $N = 75$

(corresponding to 720 ms of tracking time), resulting in a critical value for γ of 0.228, as shown in Fig. S9.

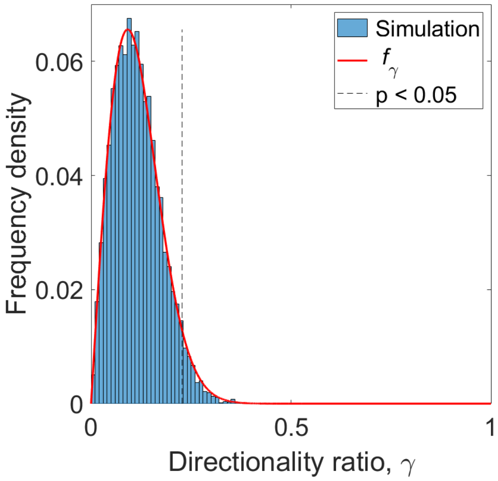

Fig. S9: Extraction of critical dimensionality ratio value. For $N = 75$, the directionality ratio of a particle undergoing Brownian motion in two dimensions at each time step over 10000 increments was simulated (blue bars) and matched with the probability density function, f_γ as derived in the text. The critical value of 0.228 corresponds to the 95% confidence interval for statistical significance.

When identifying the trajectories of the nanodiamonds that were considered to be directed motion in cells, a length threshold of 500 nm was also used to remove displacements due to movement around or induced by organelles (for example the remodelling of the mitochondrial network).

Supporting Information Section 12:

Complex modulus analysis for nocodazole-treated cells

Using the trajectories of nanodiamonds in HeLa cells subjected to a 1 hour treatment of 50 μM nocodazole, the intracellular material properties were investigated using the complex modulus, G^*. In Fig. S10, the average elastic and viscous components of G^* (G' and G'' respectively) are shown, with the elastic component dominating.

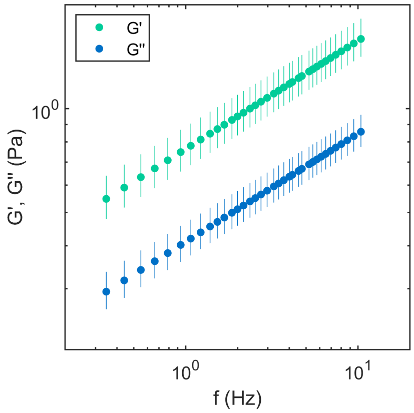

Fig. S10: Complex modulus for nanodiamonds in nocodazole-treated cells, showing that the cytoplasm displays elasticity-dominated behavior.

References

(1) Dupont, A.; Gorelashvili, M.; Schüller, V.; Wehnekamp, F.; Arcizet, D.; Katayama, Y.; Lamb, D.; Heinrich, D. Three-dimensional single-particle tracking in live cells: news from the third dimension. *New journal of physics* **2013**, *15*, 075008.

(2) Lanzanò, L.; Gratton, E. Orbital single particle tracking on a commercial confocal microscope using piezoelectric stage feedback. *Methods and applications in fluorescence* **2014**, *2*, 024010.

(3) Li, B.; Dou, S.-X.; Yuan, J.-W.; Liu, Y.-R.; Li, W.; Ye, F.; Wang, P.-Y.; Li, H. Intracellular transport is accelerated in early apoptotic cells. *Proceedings of the National Academy of Sciences* **2018**, *115*, 12118–12123.

(4) Sotoma, S.; Zhong, C.; Kah, J. C. Y.; Yamashita, H.; Plakhotnik, T.; Harada, Y.; Suzuki, M. In situ measurements of intracellular thermal conductivity using heater-thermometer hybrid diamond nanosensors. *Science advances* **2021**, *7*, eabd7888.

(5) Simpson, D. A.; Morrisroe, E.; McCoey, J. M.; Lombard, A. H.; Mendis, D. C.; Treussart, F.; Hall, L. T.; Petrou, S.; Hollenberg, L. C. Non-neurotoxic nanodiamond probes for intraneuronal temperature mapping. *ACS nano* **2017**, *11*, 12077–12086.

(6) Fujiwara, M.; Sun, S.; Dohms, A.; Nishimura, Y.; Suto, K.; Takezawa, Y.; Oshimi, K.; Zhao, L.; Sadzak, N.; Umehara, Y., et al. Real-time nanodiamond thermometry probing in vivo thermogenic responses. *Science advances* **2020**, *6*, eaba9636.

(7) Sekiguchi, T.; Sotoma, S.; Harada, Y. Fluorescent nanodiamonds as a robust temperature sensor inside a single cell. *Biophysics and physicobiology* **2018**, *15*, 229–234.

(8) Yukawa, H.; Fujiwara, M.; Kobayashi, K.; Kumon, Y.; Miyaji, K.; Nishimura, Y.; Oshimi, K.; Umehara, Y.; Teki, Y.; Iwasaki, T., et al. A quantum thermometric sensing and analysis system using fluorescent nanodiamonds for the evaluation of living stem cell functions according to intracellular temperature. *Nanoscale Advances* **2020**, *2*, 1859–1868.

(9) Kucsko, G.; Maurer, P. C.; Yao, N. Y.; Kubo, M.; Noh, H. J.; Lo, P. K.; Park, H.; Lukin, M. D. Nanometre-scale thermometry in a living cell. *Nature* **2013**, *500*, 54–58.
